# Tumorigenicity-associated characteristics of human iPS cell lines

**DOI:** 10.1371/journal.pone.0205022

**Published:** 2018-10-04

**Authors:** Satoshi Yasuda, Shinji Kusakawa, Takuya Kuroda, Takumi Miura, Keiko Tano, Nozomi Takada, Satoko Matsuyama, Akifumi Matsuyama, Michiyo Nasu, Akihiro Umezawa, Takao Hayakawa, Hideki Tsutsumi, Yoji Sato

**Affiliations:** 1 Division of Cell-Based Therapeutic Products, National Institute of Health Sciences, Kawasaki, Japan; 2 Center for Rare Disease Research, National Institute of Biomedical Innovation, Health and Nutrition, Osaka, Japan; 3 Center for Regenerative Medicine, National Research Institute for Child Health and Development, Tokyo, Japan; 4 Pharmaceutical Research and Technology Institute, Kindai University, Osaka, Japan; 5 Central Institute for Experimental Animals, Kawasaki, Japan; 6 Department of Quality Assurance Science for Pharmaceuticals, Graduate School of Pharmaceutical Sciences, Nagoya City University, Nagoya, Japan; 7 Department of Cellular & Gene Therapy Products, Graduate School of Pharmaceutical Sciences, Osaka University, Osaka, Japan; 8 Department of Translational Pharmaceutical Sciences, Graduate School of Pharmaceutical Sciences, Kyushu University, Fukuoka, Japan; University of Minnesota Medical Center, UNITED STATES

## Abstract

Human induced pluripotent stem cells (hiPSCs) represent promising raw materials of human cell-based therapeutic products (hCTPs). As undifferentiated hiPSCs exhibit intrinsic tumorigenicity properties that enable them to form teratomas, hCTPs containing residual undifferentiated hiPSCs may cause tumor formation following transplantation. We first established quantitative and sensitive tumorigenicity testing of hiPSCs dissociated into single cells using NOD/Shi-scid IL2Rγ^null^ (NOG) mice by inhibiting apoptosis of hiPSCs with a Rho kinase inhibitor. To examine different features in tumorigenicity of various hiPSCs, 10 commonly available hiPSC lines were subjected to *in vivo* tumorigenicity testing. Transplanted hiPSC lines showed remarkable variation in tumor incidence, formation latency, and volumes. Most of the tumors formed were classified as immature teratomas. However, no signs of malignancies, such as carcinoma and sarcoma, were recognized in the tumors. Characteristics associated tumorigenicity of hiPSCs were investigated with microarray analysis, karyotype analysis, and whole exome sequencing. Gene expression profiling and pathway analysis supported different features of hiPSC lines in tumorigenicity. hiPSC lines showed chromosomal abnormalities in some lines and 61–77 variants of cancer-related genes carrying effective nonsynonymous mutations, which were confirmed in the COSMIC databases. In this study, the chromosomal abnormalities and cancer-related gene mutations observed in hiPSC lines did not lead to the malignancy of tumors derived from hiPSCs. Our results suggest that the potential tumorigenicity risk of hCTPs containing residual undifferentiated hiPSCs is dependent on not only amounts of undifferentiated hiPSCs but also features of the cell lines used as raw materials, a finding that should be considered from the perspective of quality of hCTPs used.

## Introduction

Human pluripotent stem cells (hPSCs), such as human induced pluripotent stem cells (hiPSCs) and human embryonic stem cells (hESCs), are used as the raw materials of human cell-based therapeutic products (hCTPs) due to their infinite self-renewal capacity and ability to differentiate into various cell types *in vitro*. Practically, several clinical trials and studies using hPSC-derived hCTPs have already started to treat spinal cord injuries, age-related macular degeneration, heart failure, and type I diabetes around the world [1]. However, undifferentiated hPSCs are known to possess intrinsic tumorigenicity properties and to form teratomas in immunodeficient animals. In addition, safety concerns associated with teratoma derived from hPSCs have been raised for development of hCTPs [2]. The possibility cannot be excluded that a trace amount of residual undifferentiated hPSCs in hCTPs leads to tumor development following transplantation. Thus, contamination of hCTPs with residual undifferentiated hPSCs is a potential concern in terms of certain hazardous factors as well as the quality attributes of hPSC-derived hCTPs.

The World Health Organization (WHO) Technical Report Series (TRS) No. 978 Annex 3 is the only international guideline that addresses tumorigenicity testing for medicinal products [3]. However, the *in vivo* tumorigenicity testing proposed in WHO TRS 978 covers only viable animal cells used as cell substrates for manufacturing biological products but not cell products used directly for therapy by transplantation into patients. No international guideline has been issued for tumorigenicity testing of hCTPs even though its establishment is urgently needed for the development of hCTPs.

We have previously reported the performance of *in vivo* tumorigenicity testing using severe immunodeficient NOD/Shi-scid IL2Rγ^null^ (NOG) mice for the detection of HeLa cells used to spike human mesenchymal stem cells as a model of tumorigenic cellular impurity in hCTPs [4]. This *in vivo* tumorigenicity testing using NOG mice and Matrigel serves as a highly sensitive and quantitative method to detect tumorigenic cells contained in hCTPs. On the other hand, in order to form teratomas, hPSCs generally need to be injected into immunodeficient mice in the form of clumps, as hPSCs dissociated into single cells readily undergo apoptosis [5]. Thus, tumorigenicity testing has a number of challenges with regard to transplanting defined numbers of hPSCs into mice. To overcome this issue, single dissociated hESCs were spiked into mitomycin C-treated fibroblasts for transplantation into SCID mice [6] and NOD SCID mice [7] with Matrigel, which revealed that efficient engraftment of single dissociated hPSCs in immunodeficient mice requires both feeders and a basement membrane matrix in the transplanted cell mixture. Additionally, to understand the risk of tumorigenicity of hPSC-derived hCTPs, quality attributes related to tumorigenicity of hPSCs should also be taken into consideration as well as quantitative evaluation for residual undifferentiated hPSCs in hCTPs. Although the amounts of residual hPSCs in hCTPs is appropriately controlled in the manufacturing process, any different features in the tumorigenicity of hPSCs *per se* may influence tumor formation and their potential risks resulting from residual hPSCs in hCTPs. However, different features in tumorigenicity, including benign and malignancy, and its associated quality attributes of hPSCs has not been well studied.

Here, to address the issues regarding tumorigenicity of hPSCs used as raw materials for hCTPs, we established a quantitative method for detecting hiPSCs by *in vivo* tumorigenicity testing using NOG mice. Using this method, we performed tumorigenicity testing of 10 hiPSC lines and also pathologically evaluated the tumor formed in the mice to examine difference in tumorigenicity features of the hiPSC lines. A comprehensive gene expression analysis of the transplanted hiPSC lines identified genes statistically correlated with tumorigenicity of hiPSCs for the pathway analysis. In addition, we examined the mutations observed in the hiPSC lines against the cancer-related genes shown in public databases using exome sequence analysis.

## Materials and methods

### Cell culture

hiPSC lines 201B7 [8], 253G1 [9], 409B2 [10], 454E2 [10], HiPS-RIKEN-1A, HiPS-RIKEN-2A, and HiPS-RIKEN-12A were provided by the RIKEN BRC through the Project for Realization of Regenerative Medicine and the National Bio-Resource Project of the MEXT, Japan. hiPSC lines ATCC-DYR0100 and ATCC-HYR0103 were obtained from the ATCC. The human cell line mc-iPS was purchased from System Biosciences [11]. Information for the hiPSCs used in this study is described in S1 Table. hiPSCs were first cultured on mitomycin C-treated SNL cells, a mouse fibroblast STO cell line expressing the neomycin-resistance gene cassette and LIF, in primate ES cell medium (ReproCell) supplemented with 4 ng/ml human basic fibroblast growth factor (R&D Systems). hiPSCs were then passaged onto Matrigel-coated dishes with feeder-free cell-culture medium mTeSR1 (Stem Cell Technologies) for at least three passages to remove feeder cells before transplantation into mice. Undifferentiated colonies were passaged as small clumps once in every 5–6 days using CTK dissociation solution (ReproCell) and a StemPro EZPassage device (Invitrogen). Normal human neonatal dermal fibroblasts (NHDF) were obtained from Lonza and maintained in Eagle’s minimum essential medium (Sigma), supplemented with 10% fetal bovine serum (FBS; Sigma), 2 mM GlutaMax^™^ (Gibco), 50 U/ml penicillin, and 50 μg/ml streptomycin (Nacalai Tesque). The NHDF were inactivated by treatment with 10 μg/ml mitomycin C (Sigma-Aldrich) one day before transplantation into mice. Normal human bone marrow-derived mesenchymal stem cells (hMSCs) were purchased from Lonza and cultured in MSCGM^™^ medium (Lonza). All cells were cultured in a humidified atmosphere of 5% CO_2_ and 95% air at 37 °C.

### Preparation of hiPSC suspension for transplantation

Cells to be transplanted into immunodeficient mice were prepared according to the method reported by Gropp *et al*. with modification [7]. Cultured hiPSCs were treated with CTK dissociation solution and a StemPro EZPassage device or with StemPro Accutase Cell Dissociation Reagent (Gibco) to dissociate them into smaller cell clumps or single cells, respectively. To count the cell number of clumps, we used aliquots of clump suspension and enzymatically dissociated cell clamps into single cells. Mitomycin C-treated NHDFs and hMSCs were detached and dissociated with 0.25% trypsin-EDTA solution (Gibco). The hiPSCs, and mitomycin C-treated NHDFs or hMSCs were suspended together in a 1:1 mixture of mTeSR1 medium and Matrigel (product #354234, Corning) in the presence or absence of 10 μM Y-27632 (Wako) just prior to transplantation. Various numbers of hiPSCs combined with 1 × 10^6^ mitomycin C-treated NHDFs or 1 × 10^6^ hMSCs were transplanted into one mouse.

### Tumorigenicity tests

Male NOD.Cg-*Prkdc*^*scid*^ *Il2rg*^*tm1Sug*^/Jic (NOD/Shi-scid IL2Rγ^null^; NOG) mice maintained in the Central Institute for Experimental Animals (CIEA, Kanagawa, Japan) were used for *in vivo* tumorigenicity studies. All mice were housed in polycarbonate plastic cages (W155 x D245 x H149 mm; 2–3 mice/cage) with soft bedding (GLP Aspen Bedding, CLEA Japan) in an air-conditioned room at a temperature of 23 ± 2°C and a humidity of 50 ± 10% with a 12-hr light and 12-hr dark cycle. The animals were given irradiation sterilized commercial diet (CA-1, CLEA Japan) and sterilized water filled in plastic bottles *ad libitum*. Prepared cell suspensions (100 μl) were subcutaneously injected using 1 ml syringes with a 25 G needle (Terumo) into 8-week-old mice. The mice were palpated weekly for 16 weeks to observe nodule formation at the injection site. Tumor size was assessed by external measurement of the length and width of the tumors in two dimensions using a caliper as soon as tumors reached measurable size. The tumor volume was calculated using the formula: volume (mm^3^) = 1/2 length (mm) × (width [mm])^2^. The engraftment was determined according to progressive nodule growth at the injection site. Mice were euthanized and necropsied when tumors grew greater than 10% of their body weight or when a sign of deconditioning was noted according to the categories of Scientists Center for Animal Welfare (SCAW) [12] and National Institutes of Health (NIH) Animal Research Advisory Committee Guideline (https://oacu.oir.nih.gov/animal-research-advisory-committee-guidelines). Tumor-forming capacity is defined as 50% tumor-producing dose (TPD_50_), which represents the threshold dose of cells forming tumors in 50% of the animals. TPD_50_ values and their 95% confidence intervals were calculated using the Spearman-Kärber method, as implemented in the R statistical application with the package ‘tsk’ version 1.2 (https://r-forge.r-project.org/R/?group_id=1169), at each time point. For the Spearman-Kärber method to be applicable, it is necessary to use a range of doses wide enough to include both the dose at and below which 100% of the animals will be positive and the dose at and above which 100% of the animals will be negative [13]. If each of these conditions was not fulfilled, it was assumed that the next higher (10 times) or lower (1/10) dose step (a dummy set of data) to the last one tested would have produced the predicted incidence.

The protocol of the present study was reviewed beforehand and approved by the Animal Ethics Committees of CIEA and the National Institute of Health Sciences (NIHS, Kanagawa, Japan). All animal experiments were performed according to the Ethical Guidelines for Animal Experimentation from the CIEA and the NIHS. All animals were euthanized under isoflurane inhalation anesthesia.

### Histology

The resulting tumors were dissected and fixed with 10% neutral buffered formalin. The paraffin-embedded tissues were sliced into 5 μm sections and stained with hematoxylin and eosin. The specimens at different depths through the tumor mass were subjected to histological analysis by certified pathologists. The formation of differentiated tumors comprising three germ layers was evaluated, and immature teratomas were graded from 0 to 3 on the basis of the amount of immature neuroepithelium found in the tumor specimen according to the system developed by O’Connor and Norris [14].

### GeneChip and statistical analysis

Total RNA was isolated from hiPSCs using an RNeasy Mini Kit with DNase I treatment (QIAGEN) and converted to biotinylated cRNA using a GeneChip 3´ IVT Express kit (Affymetrix). Labeled RNA was processed for microarray hybridization to a GeneChip Human Genome U133 Plus 2.0 Array (Affymetrix), which contains 54,675 probe sets (~47,400 transcripts). An Affymetrix GeneChip Fluidics Station was used to perform streptavidin/phycoerythrin staining. The hybridization signals on the microarray were scanned using a GeneChip Scanner 3000 (Affymetrix) and analyzed using Expression Console Software ver.1.1 (Affymetrix). Normalization was performed using MAS5 algorithm, and the MAS5 data were scaled to a Target Intensity of 500. The hybridization experiments were performed on 3 samples of each hiPSC line. The NCBI GEO accession number for the microarray data is GSE108566.

To extract the informationally significant probe sets from the data set, we filtered probe sets using the following two steps. First, probe sets were regarded as “absent” not when indicated as “present” by “absolute analysis” using GCOS software in all 30 samples. Probe sets regarded as “absent” were eliminated from the data set. Secondly, when no significant difference was observed among cell lines using ANOVA (*p ≥* 0.05), probe sets were also eliminated from the data set. Tumorigenicity of each hiPSC line was evaluated by the latency of tumor formation and by tumor incidence. To identify probe sets related to the tumorigenicity of the hiPSC lines, the correlation between the intensity values of the filtered probe sets and the two variables (latency of tumor formation and tumor incidence) was determined by calculating Spearman’s rank correlation coefficients and their *p*-values [15]. Probe sets exhibiting statistically significant correlations with both of two variables (*p* < 0.01) were selected. The resulting probe sets were analyzed with Ingenuity Pathway Analysis software (Ingenuity Systems).

### Karyotype analysis

Chromosomal G-band analyses of hiPSCs were performed at the Nihon Gene Research Laboratories (Sendai, Japan). At least 20 metaphase spreads were examined for each cell line.

### Exome sequencing analysis

Whole exome sequencing was performed by Takara Bio Inc. Genomic DNA was isolated from hiPSCs using a NucleoSpin Tissue Kit (Macherey-Nagel). DNA (3 μg) was sheared into peak fragment size of 300 bp using a Covaris S2 sonicator (Covaris) and used to conduct end-repair, A-addition, and adaptor ligation with SureSelect XT reagents (Agilent Technologies). Target sequences were enriched with SureSelect XT Human All Exon Kit V5 (Agilent Technologies). Sequencing was performed on a HiSeq 2500 System (Illumina) using a 100 bp paired-end protocol. More than 15 Gbp of read data were generated for each sample of hiPSC line. After trimming the adaptor sequences and quality filtering using Trimmomatic [16], the reads were mapped to the reference genome (hg 19) using BWA-MEM [17], followed by duplicate read removal. Genomic variants, including SNV and short indels, were annotated using SnpEff [18], Human genetic variation database (HGVD) [19] and the Exome Aggregation Consortium (ExAC) database [20], and related to a gene list including the Cancer Gene Census provided in the COSMIC database (S2 Table). The NCBI Sequence Read Archive (SRA) accession number for the whole exome sequencing data is SRP134676.

## Results

### The *in vivo* tumorigenicity tests reflecting quantification of hiPSCs

**Fig 1:**
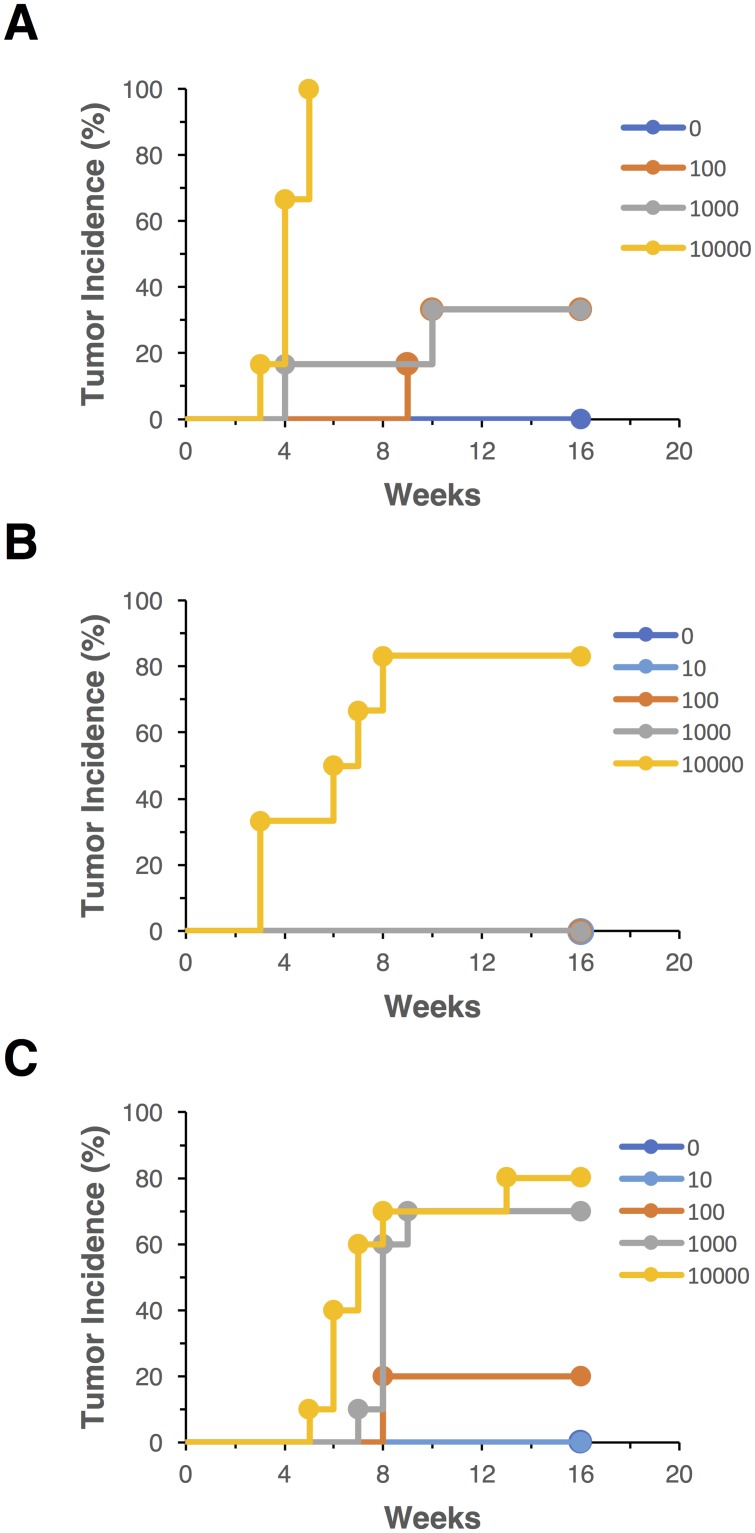
Tumor incidence of the 201B7 hiPSC line. 201B7 cells were subcutaneously injected into NOG mice at the indicated doses (0, 1 × 10^2^, 1 × 10^3^, or 1 × 10^4^ cells) with Matrigel. The 201B7 cells were transplanted as clumps (A), single cells with 1 × 10^6^ mitomycin C-treated normal human neonatal dermal fibroblasts (NHDF) (B), or single cells with 1 × 10^6^ mitomycin C-treated NHDF and 10 μM Y-27632 (C). Tumor formation was examined for 16 weeks. Six (A, B) or ten (C) mice were used in each group.

The tumorigenicity of hiPSC line 201B7 was first evaluated when these cells were transplanted into NOG mice as clumps with Matrigel. The hiPSC suspension was subcutaneously injected into NOG mice at a dose of 0, 1 × 10^2^, 1 × 10^3^, and 1 × 10^4^, and tumor formation was monitored at the transplanted sites for 16 weeks. Transplantation of 1 × 10^2^, 1 × 10^3^, and 1 × 10^4^ hiPSCs caused tumor formation within 10 weeks at an incidence of 33% (2/6 animals), 33% (2/6 animals), and 100% (6/6 animals), respectively, whereas no mice showed any tumor formation at a dose of 0 hiPSCs (Fig 1A). The TPD_50_ value was calculated as 681 when hiPSCs were transplanted into NOG mice as clumps in Matrigel (Table 1). On the other hand, to precisely define the number of hiPSCs transplanted into mice, single cell dissociation of hiPSCs would be required for cell counting. Gropp *et al*. reported a defined number of dissociated hESCs should be subcutaneously transplanted with mitotically inactivated feeder cells and Matrigel into NOD/SCID mice for a quantitative and sensitive teratoma assay [7]. Thus, we attempted to transplant single dissociated hiPSCs into NOG mice to ensure quantitativity of the tumorigenicity test. The 201B7 hiPSCs dissociated into single cells (0, 1 × 10, 1 × 10^2^, 1 × 10^3^, and 1 × 10^4^) were mixed with mitomycin C-treated NHDFs, 1 × 10^6^) and subcutaneously transplanted into NOG mice with Matrigel. Tumors formed in 83% mice (5/6 animals) at a dose of 1 × 10^4^ hiPSCs. On the other hand, no mice showed tumor formation at doses of 0, 1 × 10, 1 × 10^2^, or 1 × 10^3^ hiPSCs (Fig 1B). Its TPD_50_ value was calculated as 4642 and approximately 6.8-fold higher than that when transplanted with clumps of hiPSCs (Table 1). These results indicated that single cell dissociation of hiPSCs impairs their engraftment at the subcutaneous sites of NOG mice even in the presence of fibroblasts. Y-27632, a Rho kinase inhibitor, is well known to effectively inhibit dissociation-induced apoptosis of human pluripotent stem cells [5]. Next, using Y-27632, we performed tumorigenicity tests of hiPSCs dissociated into single cells. Single dissociated hiPSCs (0, 1 × 10, 1 × 10^2^, 1 × 10^3^, and 1 × 10^4^) were mixed with 1 × 10^6^ of mitotically inactivated NHDFs and Matrigel in the presence of 10 μM Y-27632. The prepared cell suspension was subcutaneously transplanted into NOG mice, and their tumor formation was monitored for 16 weeks. The tumor incidence of NOG mice transplanted with 1 × 10, 1 × 10^2^, 1 × 10^3^, and 1 × 10^4^ hiPSCs was 0% (0/10 animals), 20% (2/10 animals), 60% (6/10 animals), and 70% (7/10 animals), respectively (Fig 1C). The TPD_50_ value was calculated as 631 and effectively improved by mixing with Y-27632 (Table 1). We next examined subcutaneous tumor formation of hiPSCs (0, 1 × 10, 1 × 10^2^, 1 × 10^3^, and 1 × 10^4^ cells) spiked into 1 × 10^6^ hMSCs instead of NHDFs with Matrigel and Y-27632 in NOG mice. Even when injected with 1 × 10^4^ hiPSCs, no mice showed formation of tumors derived from hiPSCs in the presence of hMSCs (S3 Table). These results suggested that hMSCs suppress tumor formation of hiPSCs under some defined condition *in vivo*. Thus, we have established *in vivo* tumorigenicity tests reflecting the quantification of hiPSCs to transplant mixtures of fibroblasts, Matrigel, and a Rho kinase inhibitor into NOG mice.

**Table 1:** Tumor formation capacity of 201B7 hiPSCs in NOG mice.

Group	Tumor incidence at indicated hiPSC dose at 16 wk						TPD_50_	95% confidence interval for the TPD_50_	
	0	1 × 10	1 × 10^2^	1 × 10^3^	1 × 10^4^	1 × 10^5^		Lower	Upper
**hiPSC clumps**	0/6^a^	— (0/6)^b^	2/6	2/6	6/6	—	681	199	2327
**Single hiPSCs/NHDF**	0/6	0/6	0/6	0/6	5/6	— (6/6)^c^	4642	2336	9223
**Single hiPSCs/NHDF + Y27632**	0/10	0/10	2/10	7/10	8/10	— (10/10)^c^	631	223	1783

—: not tested

^a^Number of mice in which tumor formed/total number of mice inoculated.

^b^Since not all mice inoculated with the lowest dose (1x10^2^) showed the negative (non-tumor bearing) results, it was assumed that the tumor incidence of mice at an even lower dose step (a dummy set of data) would have been 0% for the Spearman-Kärber method to be applicable.

^c^Since not all mice inoculated with the highest dose (1x10^4^) formed tumors, it was assumed that the tumor incidence of mice at an even higher dose step (a dummy set of data) would have been 100% for the Spearman-Kärber method to be applicable.

### Difference in tumorigenic potential of 10 hiPSC lines

**Fig 2:**
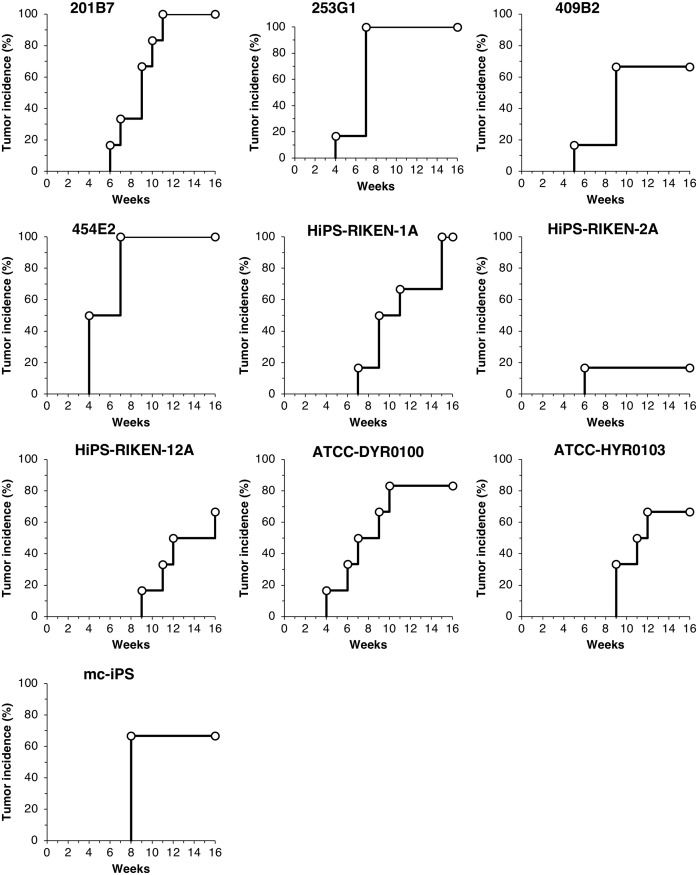
Tumor incidence of mice subcutaneously injected with 10 hiPSC lines. Dissociated single cells of hiPSC lines (201B7, 253G1, 409B2, 454E2, HiPS-RIKEN-1A, HiPS-RIKEN-2A, HiPS-RIKEN-12A, DYR0100, HYR0103, and mc-iPS) were subcutaneously transplanted into NOG mice at 3 × 10^4^ cells with Matrigel and 1 × 10^6^ mitomycin C-treated NHDF in the presence of 10 μM Y-27632. Tumor formation was examined for 16 weeks. Six mice were used in each group.

**Fig 3:**
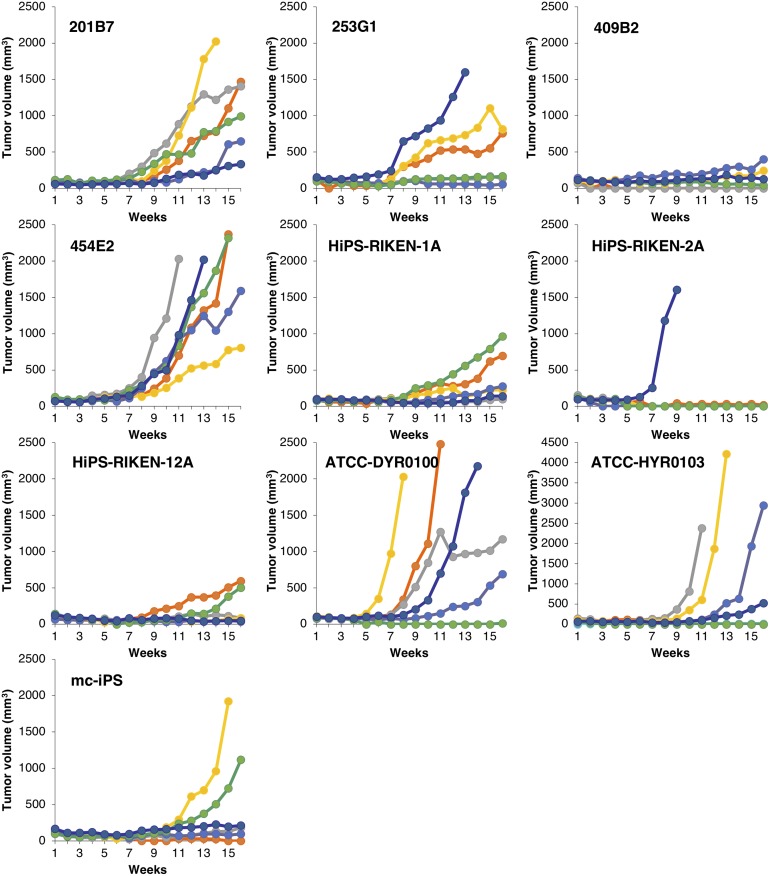
Time course of tumor volume in mice subcutaneously injected with 10 hiPSC lines. Dissociated single cells of hiPSC lines (201B7, 253G1, 409B2, 454E2, HiPS-RIKEN-1A, HiPS-RIKEN-2A, HiPS-RIKEN-12A, DYR0100, HYR0103, and mc-iPS) were subcutaneously transplanted into NOG mice at 3 × 10^4^ cells with Matrigel and 1 × 10^6^ mitomycin C-treated NHDF in the presence of 10 μM Y-27632. Tumor size was observed for 16 weeks. Six mice were used in each group.

To examine the difference in tumorigenic potential of 10 hiPSC lines (201B7, 253G1, 409B2, 454E2, HiPS-RIKEN-1A, HiPS-RIKEN-2A, HiPS-RIKEN-12A, ATCC-DYR0100, ATCC-HYR0103, mc-iPS), 3 × 10^4^ of single dissociated hiPSCs were inoculated at subcutaneous sites of NOG mice with 1 × 10^6^ of mitotically inactivated NHDFs and Matrigel in the presence of Y-27632. We continuously monitored their tumor formation for 16 weeks to obtain the data of tumor incidence, latency, and growth. The ratio of the number of mice showing tumor formation to that of their group determined tumor incidence, following 16 weeks of observation. Tumor incidence of each hiPSC line was 100% (201B7), 100% (253G1), 67% (409B2), 100% (454E2), 100% (HiPS-RIKEN-1A), 17% (HiPS-RIKEN-2A), 67% (HiPS-RIKEN-12A), 83% (ATCC-DYR0100), 67% (ATCC-HYR0103), and 67% (mc-iPS), as shown in Fig 2. With regard to tumor latency, we found symptoms of tumor formation within 9 weeks in at least one mouse in each hiPSC line group. The tumor latency of each hiPSC line was 6 weeks (201B7), 4 weeks (253G1), 5 weeks (409B2), 4 weeks (454E2), 7 weeks (HiPS-RIKEN-1A), 6 weeks (HiPS-RIKEN-2A), 9 weeks (HiPS-RIKEN-12A), 4 weeks (ATCC-DYR0100), 9 weeks (ATCC-HYR0103), and 8 weeks (mc-iPS). These results indicated that tumor incidence and latency distinctly varied in the transplanted hiPSC lines. Tumor sizes increased almost in a time-dependent manner, and growth rates of tumor mass were quite different among hiPSC lines as well as individual mice (Fig 3). Engrafted tumors of 454E2, ATCC-DYR0100, and ATCC-HYR0103 hiPSCs grew progressively to the size of more than 800, 600, and 500 mm^3^, respectively. In contrast, none of the tumors derived from 409B2, HiPS-RIKEN1A, or HiPS-RIKEN12A hiPSCs exceeded 500, 1000, or 600 mm^3^, respectively, even after 16 weeks of observation. The growth rates of tumors derived from 201B7, 253G1, and mc-iPS hiPSCs appeared to be moderate compared with the other hiPSC lines indicated above. Exclusively, rapid growth of only one tumor was seen, and no other tumor was formed in the HiPS-RIKEN-2A hiPSC group. Indeed, tumor sizes of 454E2, ATCC-DYR0100, and ATCC-HYR0103 hiPSCs began to show statistically significant differences from those of the three hiPSC groups, 409B2, HiPS-RIKEN1A, and HiPS-RIKEN12A, at 11, 11, and 13 weeks of transplantation, respectively, using two-way repeated measures ANOVA followed by Bonferroni t-test. Taken together, these results suggest that tumorigenic potential is largely dependent on the property of each hiPSC line.

### Histological analysis of formed tumor

**Fig 4:**
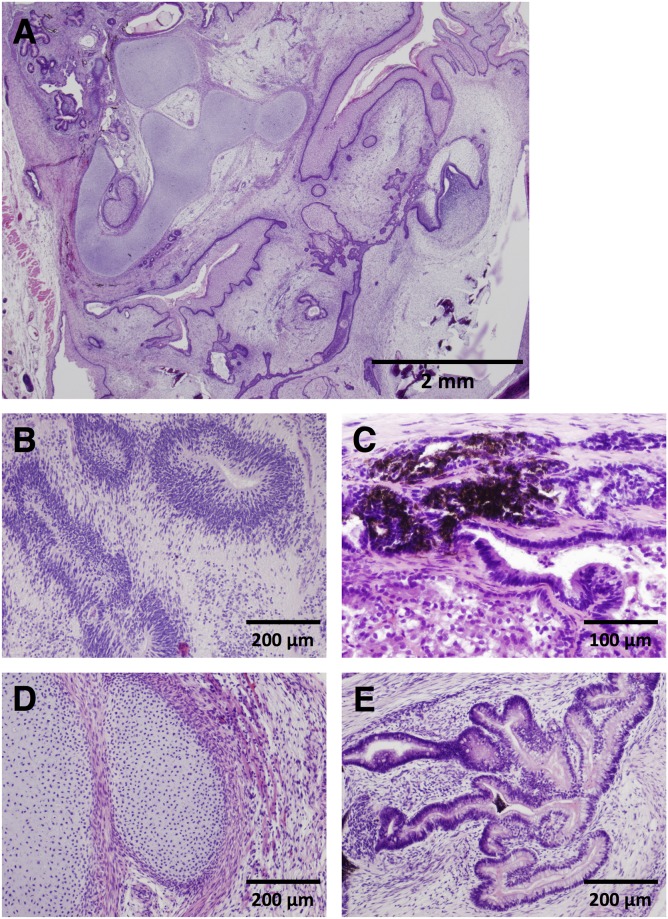
Representative images of teratomas subcutaneously formed in NOG mice injected with hiPSCs in a mixture of Matrigel, mitomycin C-treated NHDF, and 10 μM Y-27632. Excised tumor samples were stained with hematoxylin and eosin. Low power view of teratoma (A), ectodermal neuroepithelia (B), melanocytes (C), mesodermal cartilage (D), and endodermal intestinal tract-like ducts (E).

To observe tumors formed in NOG mice transplanted with the 10 hiPSC lines, we prepared tumor samples isolated from all mice showing tumor formation for histological analysis. Evaluating these H&E stained specimens, we focused on three points: a) differentiation to more than one of three germ layers (endoderm, mesoderm, and ectoderm) to define the tumor sample as a teratoma, b) grading based on the amount of neuroepithelial cell component immaturity [14], and c) formation of carcinoma and sarcoma involved in malignant tumor. Fig 4 shows typical images of teratoma with ectodermal neuroepithelia and melanocytes, mesodermal cartilage, and endodermal intestinal tract-like ducts. The observed tumor samples clearly showed differentiation into either two germ layers (mesoderm and ectoderm) or three germ layers, indicating that all of the tumor samples are first defined as teratoma (Table 2, S1 Fig). Next, we examined whether the tumor samples were categorized into immature or mature teratoma. No immature neuroepithelial cell components were found in only two samples derived from the mc-iPS line out of 47 tumor samples. Therefore, almost all of the tumor samples (45 samples) were recognized as immature teratomas, and each sample was graded from 1 to 3 (Table 2). However, we did not find any significant difference in the grades among hiPSC line groups using the Kruskal–Wallis test. In addition, neither carcinoma nor sarcoma was histopathologically observed in tumors derived from any of the hiPSC lines.

**Table 2:** Sizes and grades of tumor derived from hiPSCs transplanted into NOG mice.

hiPSC lines (passages)	No.	Tumor size (mm^3^)	Time after transplantation (wk)	Differentiation into germ layers	Grade of teratoma
201B7 (p35)	1	1468	16	Three germ layers	3
	2	1406	16	Three germ layers	1
	3	2026	14	Three germ layers	3
	4	648	16	Three germ layers	1
	5	991	16	Ectoderm/mesoderm	3
	6	332	16	Three germ layers	1
253G1 (p46)	1	760	16	Three germ layers	1
	2	139	16	Ectoderm/mesoderm	1
	3	814	16	Ectoderm/mesoderm	1
	4	58	16	Three germ layers	1
	5	168	16	Ectoderm/mesoderm	1
	6	1599	13	Ectoderm/mesoderm	3
409B2 (p38)	3	244	16	Ectoderm/mesoderm	2
	4	400	16	Ectoderm/mesoderm	3
	5	43	16	Three germ layers	1
	6	128	16	Ectoderm/mesoderm	1
454E2 (p44)	1	2369	15	Three germ layers	3
	2	2030	11	Three germ layers	1
	3	804	16	Ectoderm/mesoderm	1
	4	1592	16	Ectoderm/mesoderm	1
	5	2319	15	Three germ layers	1
	6	2021	13	Three germ layers	1
HiPS-RIKEN-1A (p24)	1	697	16	Three germ layers	3
	2	99	16	Three germ layers	1
	3	226	16	Ectoderm/mesoderm	3
	4	273	16	Three germ layers	2
	5	961	16	Three germ layers	1
	6	141	16	Three germ layers	1
HiPS-RIKEN-2A (p27)	6	1603	9	Three germ layers	2
HiPS-RIKEN-12A (p32)	1	595	16	Ectoderm/mesoderm	1
	2	85	16	Three germ layers	1
	5	506	16	Three germ layers	3
	6	49	16	Three germ layers	1
ATCC-DYR0100 (unknown; >p11)	1	2481	11	Three germ layers	3
	2	1172	16	Three germ layers	1
	3	2030	8	Three germ layers	1
	4	690	16	Three germ layers	1
	6	2179	14	Three germ layers	1
ATCC-HYR0103 (unknown; >p10)	2	2380	11	Three germ layers	1
	3	4218	13	Three germ layers	2
	4	2950	16	Three germ layers	2
	6	524	16	Three germ layers	1
mc-iPS (unknown; >p27)	2	181	16	Three germ layers	0
	3	1921	15	Three germ layers	0
	5	1115	16	Three germ layers	3
	6	213	16	Three germ layers	1

### Gene expression correlated with tumorigenicity of hiPSC lines

Next, we attempted to find marker genes, the transcript expression of which in hiPSCs is correlated with their tumorigenicity. We examined their transcriptional profiles using microarray analysis, and the defined filtering criteria (see Material and methods) identified a set of 16,454 probes with significantly different expression levels among 10 hiPSC lines (S2 Fig). To isolate genes associated with tumorigenicity of the hiPSC lines, we compared GeneChip signal intensities of the hiPSC lines with the latency of tumor formation and tumor incidence in NOG mice transplanted with these cells as described in the Materials and Methods section. The correlations of signal intensities of the filtered probe sets with two variables, i.e., (i) latency of tumor formation and (ii) tumor incidence, were determined by calculating Spearman’s rank correlation coefficients: The rank order of tumor formation latency, 253G1 = 454E2 = ATCC-DYR0100 < 409B2 < 201B7 = HiPS-RIKEN-2A < HiPS-RIKEN-1A < mc-iPS < HiPS-RIKEN-12A = ATCC-HYR0103; the rank order of tumor incidence, HiPS-RIKEN-2A < 409B2 = HiPS-RIKEN-12A = ATCC-HYR0103 = mc-iPS < ATCC-DYR0100 < 201B7 = 253G1 = 454E2 = HiPS-RIKEN-1A. Finally, 289 resulting probe sets derived from 258 genes exhibited statistically significant positive or negative correlation (*p* < 0.01) with both two variables (S4 Table). The expression of pluripotency markers, OCT3/4, NANOG, SOX2, and LIN28A, did not exhibit significant correlation with tumor formation latency and incidence, and the integration of reprogramming factor genes into genome of hiPSCs did not also affect the transcript expression (S3 Fig). Pathway analysis using these genes showed top 5 networks (S5 Table), and the top diseases and functions included cellular assembly and organization, developmental disorder, and hematological diseases. In addition, 5-fluorouracil (5-FU), LIN28A, let-7, RASSF5, and ALKBH5 were shown as top 5 upstream regulators, all of which have been reported to be related to tumor formation (Table 3). These results suggest that the networks of our identified genes would play central roles in the *in vivo* tumorigenicity of hiPSCs.

**Table 3:** Top 5 of upstream regulators.

Upstream Regulator	Genes	*p*-value of overlap
5-fluorouracil	ATP5C1, ATP5O, CCT4, GTF2I, HNRNPAB, IARS, RPS16, RPS4X, SMS, UNG, YAP1	4.12 × 10^−4^
LIN28A	CDC20, CDC25A, TIA1	1.53 × 10^−3^
let-7	CDC16, CDC20, CDC25A, E2F5, GTF2I, MCM7, YAP1	5.89 × 10^−3^
RASSF5	LSM7, NAP1L3, PSMD1	6.36 × 10^−3^
ALKBH5	ADGRG2, CELSR2, CLDN11, PAFAH1B1, PRDX4	6.49 × 10^−3^

### Karyotype of transplanted hiPSC lines

**Fig 5:**
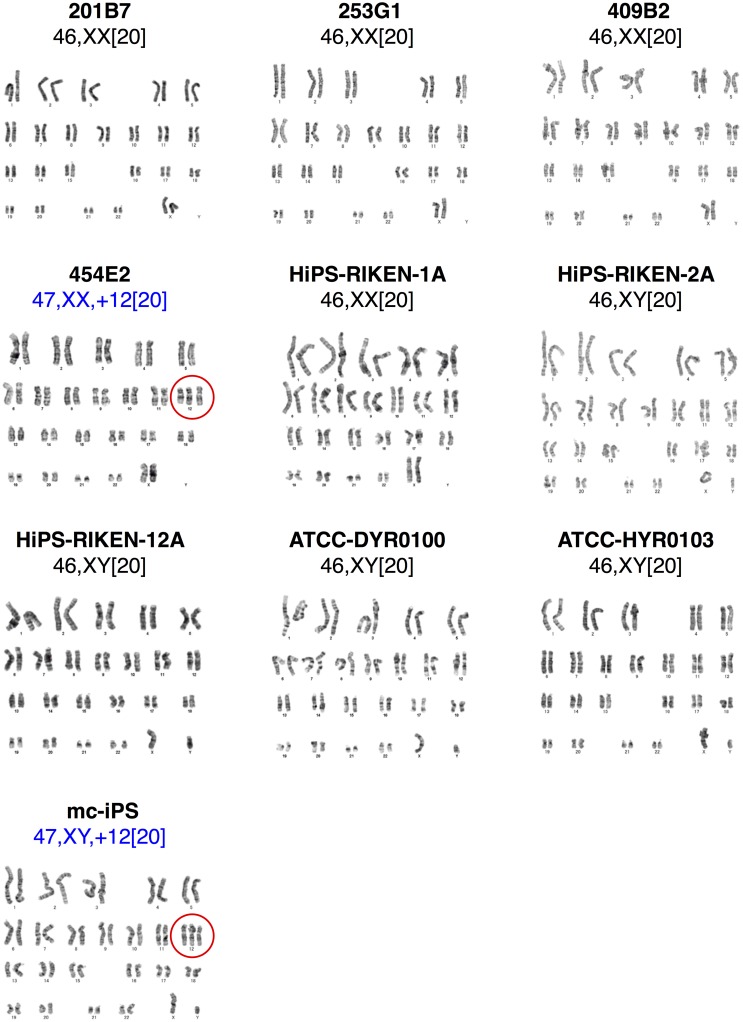
Karyotype analysis of the 10 hiPSC lines. *In vitro* cultured hiPSC lines (201B7, 253G1, 409B2, 454E2, HiPS-RIKEN-1A, HiPS-RIKEN-2A, HiPS-RIKEN-12A, DYR0100, HYR0103, and mc-iPS) were subjected to karyotype analysis. 454E2 and mc-iPS lines showed trisomy 12. Chromosomal aberrations are encircled.

Karyotype analysis is frequently used for quality assessment of PSCs to examine chromosomal abnormalities in structure and number [21]. According to the karyotype analysis of the 10 hiPSC lines cultured under our conditions for transplantation into mice, a normal karyotype was observed in 8 hiPSC lines but not in 2 hiPSC lines (mc-iPS and 454E2). Both of these hiPSC lines exhibited the same type of chromosomal abnormality, trisomy 12, in all tested cells (Fig 5). It is likely that hiPSCs having trisomy 12 exclusively grew *in vitro* because trisomy 12 has been reported to increase proliferation rates of hPSCs [22]. However, we did not observe distinct effects of trisomy 12 on the tumorigenicity potential of hiPSCs and histological properties of hiPSC-derived tumors.

### Exome sequence analysis of cancer-related genes in hiPSCs

The analysis of mutations in cancer-related genes has recently been applied in the quality evaluation of hiPSC lines using next-generation sequencing (NGS). We, therefore, performed exome sequence analysis and examined SNV and indel mutations of 613 cancer-related genes publically shown in databases including the COSMIC Cancer Gene Census. These 613 cancer-related genes are listed in S2 Table. A mutation rate of greater than 9% is considered to be reliable with more than 15 Gb read data per sample under the condition used in this exome sequencing analysis (Miura *et al*., unpublished observation). Therefore, we first searched for mutations with a mutation rate of more than 9% in cancer-related genes. Second, read depth was set at 40 or more than 40, which covered more than 97.3% of the target sequences in all analyzed samples. Third, we focused on mutations that would highly or moderately impact the amino acid sequence of the encoding protein in cancer-related genes as indicated by the possible functional change of the translated proteins [18]. Fourth, we further extracted gene mutations as classified into variants of COSMIC database. We identified 61–77 variants of cancer-related genes that satisfy the criteria per hiPSC line (Table 4, S6 Table). Mutation profiles of 201B7, 253G1, and 409B2 hiPSC lines were quite similar, likely due to the common fibroblasts used for establishing hiPSCs. The allele frequency reported in public exome sequencing database, HGVD and ExAC, was exhibited on the mutations observed in hiPSCs in the COSMIC database (S7 Table). Our findings showed that the identified mutations in the indicated cancer-related genes did not directly lead to malignancy of the teratomas derived from the hiPSCs under our experimental conditions.

**Table 4:** Genes having mutations, which were assessed as a high or moderate impact and confirmed in the COSMIC cancer database.

hiPSC lines	Genes having mutations reported in the COSMIC database
201B7	TNFRSF14, SPEN, NOTCH2, PIK3CA, TET2, FAT1, TERT, DROSHA, IL7R, APC, HLA-A*, NFKBIE, KMT2C, PCM1, NCOA2, OMD, NOTCH1, KAT6B, **CREB3L1**, MEN1, MAML2, ATM, KCNJ5, PTPRB, SH2B3, HNF1A, NCOR2, FLT3, ERCC5, FOXA1, HIF1A, ZFHX3, TP53, BRCA1, AXIN2, SETBP1, MAP2K2, JAK3, EP300, ZRSR2, FANCB
253G1	TNFRSF14, SPEN, NOTCH2, PIK3CA, TET2, FAT1, TERT, DROSHA, IL7R, APC, **HLA-A**, NFKBIE, CREB3L2, KMT2C, PCM1, NCOA2, OMD, NOTCH1, KAT6B, **CREB3L1**, MEN1, MAML2, ATM, KCNJ5, PTPRB, SH2B3, HNF1A, NCOR2, FLT3, ERCC5, FOXA1, HIF1A, ZFHX3, TP53, BRCA1, AXIN2, SETBP1, JAK3, EP300, AR, FANCB
409B2	TNFRSF14, SPEN, **NOTCH2**, PIK3CA, TET2, FAT1, TERT, DROSHA, IL7R, APC, **HLA-A**, NFKBIE, CREB3L2, KMT2C, PCM1, NCOA2, OMD, NOTCH1, KAT6B, **CREB3L1**, MEN1, MAML2, ATM, KCNJ5, PTPRB, SH2B3, HNF1A, NCOR2, FLT3, ERCC5, FOXA1, HIF1A, ZFHX3, TP53, BRCA1, AXIN2, SETBP1, JAK3, EP300, AR, FANCB
454E2	**NOTCH2**, ALK, AFF3, **FANCD2**, ATR, **WWTR1**, TET2, FAT4, FAT1, **SDHA**, IL6ST, APC, RANBP17, NSD1, NFKBIE, EGFR CREB3L2, EZH2, KMT2C, FANCG, NCOA4, KAT6B, FGFR2, **CREB3L1**, MEN1, MAML2, ATM, KCNJ5, ZNF384, SH2B3, HNF1A, NCOR2, ERCC5, FOXA1, ZFHX3, TP53, ERBB2, RNF43, RNF43, AXIN2, SETBP1, KDM6A, ATRX
HiPS-RIKEN-1A	SPEN, **NOTCH2**, ALK, **FANCD2**, FOXL2, KIT, KDR, PTPN13, TET2, FAT4, FAT1, SDHA, IL7R, APC, NSD1, **HLA-A**, NFKBIE, TRRAP, CREB3L2, KMT2C, NCOA2, CDKN2A, FANCG, PTCH1, KAT6B, FGFR2, **CREB3L1**, MEN1, MAML2, ATM, CBL, KCNJ5, ZNF384, SH2B3, HNF1A, NCOR2, **FLT3**, BRCA2, ERCC5, FOXA1, BLM, ZFHX3, ERBB2, RNF43, AXIN2, SETBP1, AR, ATRX
HiPS-RIKEN-2A	TNFRSF14, SPEN, **NOTCH2**, ALK, **FANCD2**, FOXL2, ATR, **WWTR1**, KDR, TET2, FAT4, FAT1, **SDHA**, APC, RANBP17, **HLA-A**, EGFR, CREB3L2, KMT2C, PTCH1, KAT6B, FGFR2, **CREB3L1**, MEN1, MAML2, ATM, KCNJ5, ZNF384, COL2A1, SH2B3, HNF1A, NCOR2, FLT3, BRCA2, ERCC5, FOXA1, ZFHX3, TP53, NF1, BRCA1, RNF43, AXIN2, SETBP1, TCF3, KDM6A, ATRX
HiPS-RIKEN-12A	TNFRSF14, **NOTCH2**, ALK, **FANCD2**, GATA2, ATR, **WWTR1**, KDR, PTPN13, TET2, FAT4, FAT1, APC, RANBP17, **HLA-A**, NFKBIE, EGFR, CREB3L2, KMT2C, PTCH1, RET, KAT6B, TCF7L2, **CREB3L1**, MEN1, MAML2, ATM, KCNJ5, ZNF384, COL2A1, SH2B3, HNF1A, NCOR2, POLE, FLT3, BRCA2, ERCC5, FOXA1, HIF1A, ZFHX3, TP53, RNF43, AXIN2, STK11, TCF3, ATRX
ATCC-DYR0100	TNFRSF14, **NOTCH2**, ALK, **FANCD2**, ATR, **WWTR1**, KDR, PTPN13, TET2, FAT1, DROSHA, IL7R, APC, NSD1, **HLA-A**, ELN, CREB3L2, KMT2C, KAT6B, **CREB3L1**, MEN1, MAML2, ATM, KCNJ5, CHD4, ZNF384, SH2B3, HNF1A, NCOR2, POLE, FLT3, ERCC5, FOXA1, ZFHX3, TP53, BRCA1, RNF43, SETBP1, ASXL1, MN1, EP300
ATCC-HYR0103	TNFRSF14, SPEN, **NOTCH2**, ALK, MSH2, KDR, FAT4, FAT1, IL7R, APC, KMT2C, NRG1, PDCD1LG2, OMD, TSC1, **CREB3L1**, MEN1, MAML2, ATM, KCNJ5, ZNF384, COL2A1, KMT2D, SH2B3, HNF1A, NCOR2, FLT3, FOXA1, ZFHX3, TP53, BRCA1, RNF43, AXIN2, MN1, EP300, AR, ATRX
mc-iPS	TNFRSF14, SPEN, NOTCH2, **FANCD2**, FOXL2, ATR, KIT, KDR, PTPN13, TET2, FAT4, FAT1, IL7R, APC, **HLA-A**, CREB3L2, KMT2C, PTCH1, NOTCH1, **CREB3L1**, MEN1, MAML2, ATM, KCNJ5, CHD4, ZNF384, SH2B3, HNF1A, NCOR2, FLT3, BRCA2, FOXA1, ZFHX3, TP53, RNF43, AXIN2, SETBP1, TCF3, JAK3, KDM6A, ATRX

*Variants of gene symbols indicated in bold letters were assessed as a “high” impact by SnpEff software.

## Discussion

We have established *in vivo* testing to quantify undifferentiated hPSCs using severe immunodeficient NOG mice. When defined numbers of dissociated single cells of the hiPSC line 201B7 were mixed with feeder cells, Matrigel and a Rho kinase inhibitor and subcutaneously transplanted into NOG mice, the TPD_50_ was calculated as 631. In the tumorigenicity testing, the 10 hiPSC lines differed in tumor incidence, formation latency, and volumes, indicating variety of tumorigenicity in these hiPSC lines. Almost of the formed tumors were categorized as immature teratomas and did not exhibit any signs of carcinoma or sarcoma, although the transplanted hiPSC lines possessed mutations putatively impacting the translated structure of cancer-related genes and in part showed chromosomal aberrations. We identified genes that were expressed in the hiPSCs and that significantly correlated with tumorigenicity and found their upregulators using pathway analysis.

Regarding the methods for quantifying undifferentiated hPSCs by *in vivo* tumorigenicity testing, Gropp *et al*. previously reported the sensitive teratoma assay of single cell-dissociated hESCs with mitotically inactivated feeder cells and Matrigel using NOD/SCID mice [7]. This paper described that addition of a Rho kinase inhibitor had no effect on the timing of tumor detection or its size, which was inconsistent with our results using NOG mice. Although the reason for this discrepancy is still unknown, differences in the immunosuppression of the mouse strains used for assaying would possibly affect the efficiency of hPSC engraftment. On the other hand, in this study, no tumor formation was detected in NOG mice transplanted with the mixture of hiPSCs when hMSCs were used instead of fibroblasts. This observation suggests that hMSCs suppress the growth of hiPSCs *in vivo*, although hMSCs are well known feeder cells used for *in vitro* co-culture with hPSCs [23]. Recently, Chang *et al*. reported that human umbilical cord mesenchymal stem cells abolish *in vivo* tumorigenicity of co-cultured hESCs by suppressing their WNT/β-catenin signaling [24]. hMSCs may possibly inhibit tumor formation of hiPSCs in mice by the same mechanism as in *in vitro* culture. In this context, Kanemura *et al*. reported that retinal pigment epithelium induced apoptosis in co-cultured hiPSCs *in vitro* and also suppressed tumor formation potential of co-transplanted iPSCs *in vivo* [25], suggesting that some micro-environment constituted of cells influences the survival of hiPSCs.

The hiPSC lines used in this study are commonly available and were established with several types of primary cells and reprogramming methods. The transplanted hiPSC lines markedly varied in tumorigenicity features, including in tumor incidence, formation latency, and volumes. Thus, the capacity to form tumors is largely dependent on each hiPSC line. It seems that the tumorigenicity of residual hiPSCs contained in hCTPs is evaluated differently according to the hiPSC lines used as raw materials even if the number of residual hiPSCs is estimated to be the same as those levels used in *in vitro* methods. Histopathological analysis showed that 96% of the tumors derived from the transplanted hiPSCs were evaluated as immature teratomas composed of mature and immature tissues. Indeed, immature neuroepithelium is commonly observed in teratomas produced from hPSCs xenografted in immunodeficient mice [26]. Interestingly, Akutsu *et al*. reported that teratomas derived from hESCs histologically matured *in vivo* over time [27]. Therefore, the formation of immature teratomas *in vivo* is regarded as a quality attribute of hPSCs, and immaturity of teratomas largely depends on the duration of hPSC transplantation.

Here we extracted 258 genes, the expression of which positively and negatively correlated with the tumorigenicity properties of the hiPSCs, and pathway analysis with these genes revealed upstream regulators including transcription factors, microRNAs, and drugs. 5-fluorouracil is an analog of uracil used for the treatment of cancer. LIN28A is known to block the expression of let-7 microRNA which has tumor suppressor function. RASSF5 is a member of the Ras association domain and serves as tumor suppressor. ALKBH5 is *N*^6^-methyladenosine demethylase and recently reported to be involved in the tumorigenicity of glioblastoma stem-like cells [28–31]. These upstream regulators would reflect gene clusters constructing several central networks in the tumorigenicity of hiPSCs. Our identified genes are expected to be useful in evaluating the tumorigenicity properties of hiPSCs as the raw materials of hCTPs.

Exome and whole genome sequence analysis with NGS in the search for genetic variants is now attempted to be applied in the quality assessment of clinical-grade hPSCs as raw materials of hCTPs [32]. Using exome sequence analysis with hiPSCs, we detected approximately 150 cancer-related genes per hiPSC line having nonsynonymous mutations that were predicted to affect the function of the translated proteins, and 61–77 mutations in the cancer-related genes were confirmed in the COSMIC databases. Moreover, 2 of the 10 hiPSC lines showed chromosomal abnormality. However, in spite of the variable mutations and chromosomes in hiPSCs, no malignant signs, i.e., sarcoma and carcinoma, were observed in all of hiPSC-derived tumors. These results suggest that the assessment of mutations in cancer-related genes and chromosomal abnormalities is not so useful in predicting malignancy of hiPSCs transplanted into immunodeficient mice at present. Studies to reveal the relationship of tumor malignancy with specific cancer-related gene mutations and chromosomal abnormalities are required in future to provide cancer-related gene mutations and karyotypes for the assessment of hiPSCs as raw materials of hCTPs.

The qualification of hPSCs as raw materials is one of the issues in the development and manufacturing of hCTPs. In our experiments, mutations in cancer-related genes and chromosomal abnormalities found in hiPSC lines did not predict malignancy in these cell lines, which was confirmed by *in vivo* tumorigenicity testing. Qualification of hPSCs should be mainly discussed in terms of suitability for manufacturing the final products. Our *in vivo* tumorigenicity testing method followed by pathological evaluation will contribute to the assessment of hCTPs as well as raw materials, in terms of tumorigenic cell impurities and malignancy. Furthermore, to internationally standardize tumorigenicity testing methods for hCTPs, it would be necessary to establish standard protocols and to validate the testing methods according to reports. Hopefully, our method will support international standardization as a reference of the testing methods to detect residual undifferentiated cells of hCTPs in the future.

## Supporting information

S1 Table hiPSC lines used for tumorigenicity testing. (DOCX)

S2 Table A list of reported cancer-related genes. (XLSX)

S3 Table Tumor formation capacity of 201B7 hiPSCs with hMSCs in NOG mice. (DOCX)

S4 Table Genes correlated to tumor incidence and latency in hiPSCs. (XLSX)

S5 Table Top 5 networks of genes correlated to tumor incidence and latency. (DOCX)

S6 Table Mutations assessed as a high or moderate impact and confirmed in the COSMIC cancer database. (XLSX)

S7 Table Allele frequency in public database on COSMIC cancer mutations observed in hiPSCs. (XLSX)

S1 Fig Histological analysis of teratomas formed after transplantation of 10 hiPSC lines. NOG mice were subcutaneously injected with 3 × 10^4^ hiPSCs in a mixture of Matrigel, 1 × 10^6^ mitomycin C-treated NHDF, and 10 μM Y-27632. Typical images of teratomas derived from 10 hiPSC lines are shown stained with hematoxylin and eosin [201B7 #3 (A, B), 253G1 #6 (C, D), 409B2 #3 (E, F), 454E2 #6 (G, H), HiPS-RIKEN-1A #6 (I, J), HiPS-RIKEN-2A #6 (K, L), HiPS-RIKEN-12A #5 (M, N), ATCC-DYR0100 #6 (O, P), ATCC-HYR0103 #4 (Q, R), and mc-iPS #5 (S, T)]. Low power view (× 1.25) of teratoma represents two or three germ layer components (A, C, E, G, I, K, M O, Q and S). Higher power view (× 10) shows mesodermal cartilage and endodermal intestinal tract-like duct (B), ectodermal glial tissues, melanocytes and choroid-like tissues (D), ectodermal glial tissues (F), mesodermal smooth muscle and endodermal intestinal tract-like duct (H), ectodermal choroid-like tissues and immature neuroepithelia (J), ectodermal choroid-like tissues and melanocytes (L), ectodermal stratified squamous epithelia and endodermal duct structures (N), ectodermal glial and neural cells and mesodermal blood vessels (P), endodermal duct structures accompanied with intestinal and respiratory epithelium-like cells (R), and mesodermal smooth muscle and endodermal intestinal tract structures (T).
(PDF)

S2 Fig Hierarchical clustering analysis of gene expression in 10 hiPSC lines. A set of 16,454 probes on GeneChip Human Genome U133 Plus 2.0 Array was statistically identified with significantly different expression levels among 10 hiPSC lines (one-way ANOVA, p < 0.05). Hierarchical clustering analysis was performed using R version 3.5.1 software.
(TIFF)

S3 Fig Expression of pluripotency markers in 10 hiPSC lines. Transcript expression of OCT3/4 (A), NANOG (B), SOX2 (C), and LIN28A(D) in 10 hiPSC lines is shown with microarray data. Data are represented as mean ± SD (n = 3).
(TIFF)
