# Supplementary material for: Tumorigenicity-associated characteristics of human iPS cell lines
Source: PLoS One. 2018 Oct 4;13(10):e0205022. doi: 10.1371/journal.pone.0205022 (PMC6171902; doi:10.1371/journal.pone.0205022)
Supplement: S1 Table — (DOCX) [file pone.0205022.s001.docx]

**Table S1 hiPSC lines used for tumorigenicity testing**

| **hiPSC lines** | **Primary cell types** | **Reprograming factors** | **Transduction** |
| --- | --- | --- | --- |
| 201B7 | Skin fibroblast | OCT3/4, SOX2, KLF4, c-MYC | Retrovirus vectors |
| 253G1 | Skin fibroblast | OCT3/4, SOX2, KLF4 | Retrovirus vectors |
| 409B2 | Skin fibroblast | OCT3/4, SOX2, KLF4, l-MYC, LIN28, p53shRNA | Episomal vectors |
| 454E2 | Dental pulp cells | OCT3/4, SOX2, KLF4, l-MYC, LIN28, p53shRNA | Episomal vectors |
| HiPS-RIKEN-1A | Umbilical cord fibroblast | OCT3/4, SOX2, KLF4, c-MYC | Retrovirus vectors |
| HiPS-RIKEN-2A | Umbilical cord fibroblast | OCT3/4, SOX2, KLF4, c-MYC | Retrovirus vectors |
| HiPS-RIKEN-12A | Umbilical cord fibroblast | OCT3/4, SOX2, KLF4 | Retrovirus vectors |
| ATCC-DYR0100 | Skin fibroblast | OCT3/4, SOX2, KLF4, c-MYC | Retrovirus vectors |
| ATCC-HYR0103 | Hepatic fibroblast | OCT3/4, SOX2, KLF4, c-MYC | Retrovirus vectors |
| mc-iPS | Adipose tissue derived stem cells | OCT3/4, SOX2, NANOG, LIN28 | Plasmid vectors |
