# Supplementary material for: Tumorigenicity-associated characteristics of human iPS cell lines
Source: PLoS One. 2018 Oct 4;13(10):e0205022. doi: 10.1371/journal.pone.0205022 (PMC6171902; doi:10.1371/journal.pone.0205022)
Supplement: S3 Table — (DOCX) [file pone.0205022.s003.docx]

**Table S3 Tumor formation capacity of 201B7 hiPSCs with hMSCs in NOG mice**

| **Group** | **Tumor incidence at indicated hiPSC dose at 16 wk** | | | | | **TPD_50_** |
| --- | --- | --- | --- | --- | --- | --- |
|  | 0 | 1 x 10 | 1 x 10^2^ | 1 x 10^3^ | 1 x 10^4^ |  |
| **Single hiPSCs/hMSCs + Y27632** | 0/10^a^ | 0/10 | 0/10 | 0/10 | 0/10 | ND |

ND: not determined.

^a^Number of mice in which tumor formed/total number of mice inoculated.
