# Supplementary material for: Tumorigenicity-associated characteristics of human iPS cell lines
Source: PLoS One. 2018 Oct 4;13(10):e0205022. doi: 10.1371/journal.pone.0205022 (PMC6171902; doi:10.1371/journal.pone.0205022)
Supplement: S5 Table — (DOCX) [file pone.0205022.s005.docx]

**Table S5 Top 5 networks of genes correlated to tumor incidence and latency**

| **Molecules in Network** | **Score** | **Focus Molecules** | **Top Diseases and Functions** |
| --- | --- | --- | --- |
| ***AFG3L2,** Akt, **ATP5C1, CEP162, COBL, DIAPH2, ERC1, EXOC2, FUNDC1, GOSR2, HAUS6, IMMT, IVD, MAPRE1, METTL17, MIB1, MLPH, MRPL14, MRPL17, MRPL58, OFD1, PIN4, PRDX4, PREX2, SENP2, SLC25A4, SNTB2, SPATA5, SRXN1, STX4, TBC1D2B, TPD52, TRDMT1, TTC3, TXLNG** | 68 | 34 | Cellular Assembly and Organization, Developmental Disorder, Hematological Disease |
| 14-3-3, Alpha tubulin, APC (complex), **CBY1, CCDC88A, CCT4, CCT7, CDC16, CDC20, CDC25A,** Cyclin A, Cyclin B, Cyclin E, **DDX17, DESI2,** E2f, ERK1/2, **GNAI3, GPN1, HDAC9, HERC5, IL17RD, KCTD5,** MAP2K1/2, **MCM7, PCYT1A, PDE3A, PDK3, PLS3, POLR3G,** Rb, TCF, **TPR, TRMT112, VRK2** | 40 | 24 | Cell Cycle, Cancer, Organismal Injury and Abnormalities |
| **AIM2, Alp, ATG101, ATG4A, ATP6AP2,** BCR (complex), **BEX2, BEX3, C1R, COMMD1, DDRGK1, GTF2I,** GTPase, **HSP90B1, IARS,** IFN Beta, IgG, Igm, **Immunoglobulin, INSIG1, ITGA6, LARS,** LDL, Mek, NFkB (complex), P-TEFb**, PAFAH1B1, PDHA1, PDHX, PHLDB2,** PI3K (family), **PIM2, RDX, SMG7, UNG** | 38 | 23 | Developmental Disorder, Hereditary Disorder, Metabolic Disease |
| **ALMS1,** AMPK, Ap1**, ARF5, CFAP20,** Collagen(s), Creb, cytochrome C, **DDAH1, E2F5, EGFL6,** ERK, **FHL2, GNPDA1,** HDL, Integrin, **LAMP1, MTCH1, NLGN4X, NPLOC4, OSBPL3,** Pdgf (complex), PDGF BB, Pdgfr, **PIK3R2,** Pkc(s), **PRSS35, PSEN2, RFX3, SH3KBP1,** Sos, **TAOK1,** TCR, **TIA1, ZNF665** | 34 | 21 | Neurological Disease, Organismal Injury and Abnormalities, Hereditary Disorder |
| **AATF, BAZ1A,** CD3, **CD47, EFCAB7,** F Actin, **GON7,** Gpcr, HISTONE, Histone h3, Histone h4, **HMGB3,** IL12 (complex), IL12 (family), Interferon alpha, Jnk, **MDH2, MPHOSPH6, NAB1, NFATC2IP,** P38 MAPK, **PARP1, PAXBP1, PEG10,** PI3K (complex), **POMZP3, PSMD1,** Ras homolog, **SAMHD1, SMS,** SRC (family), **TFE3,** Tlr, **TPRKB, VPS35** | 31 | 20 | Connective Tissue Disorders, Developmental Disorder, Hereditary Disorder |

*Gene symbols indicated in bold letters were identified to be statistically correlated to tumor incidence and latency in our experiments.
