# Supplementary figures and images for: Tumorigenicity-associated characteristics of human iPS cell lines
Source: PLoS One. 2018 Oct 4;13(10):e0205022. doi: 10.1371/journal.pone.0205022 (PMC6171902; doi:10.1371/journal.pone.0205022)

**201B7**

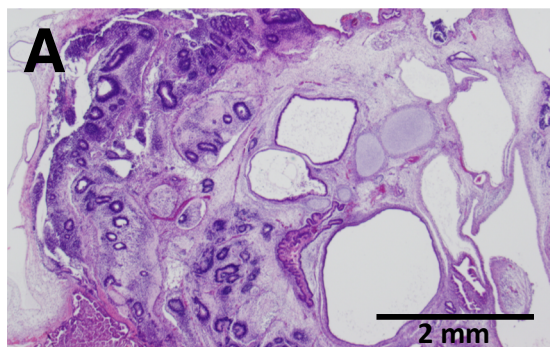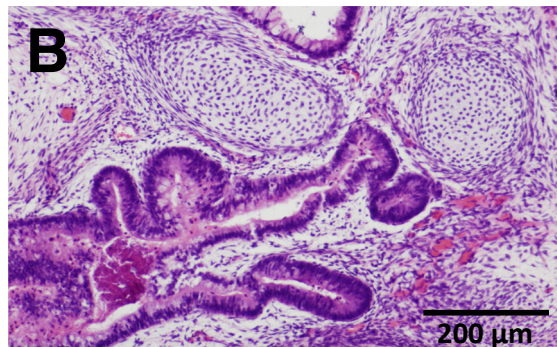

**253G1**

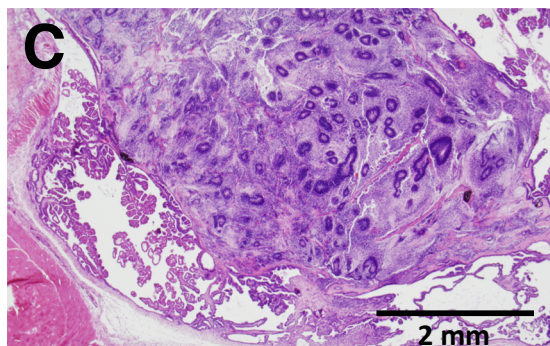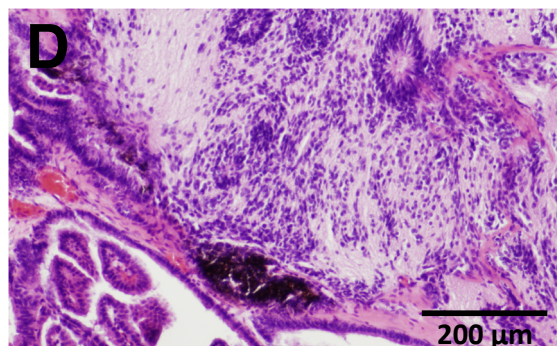

**409B2**

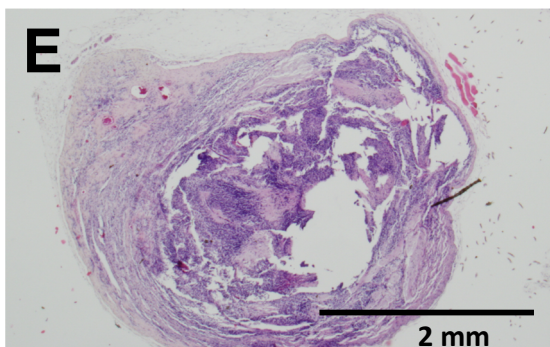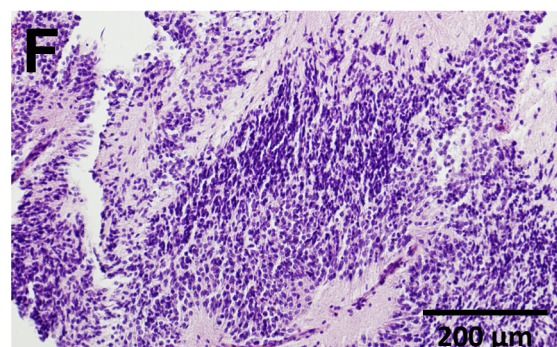

**454E2**

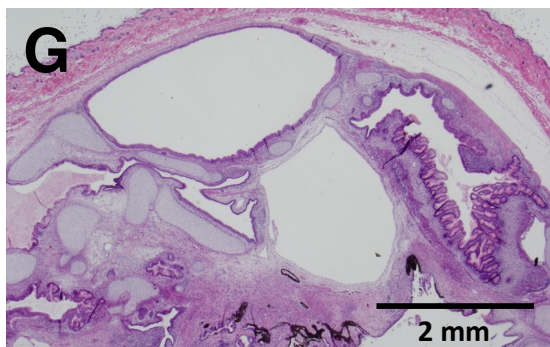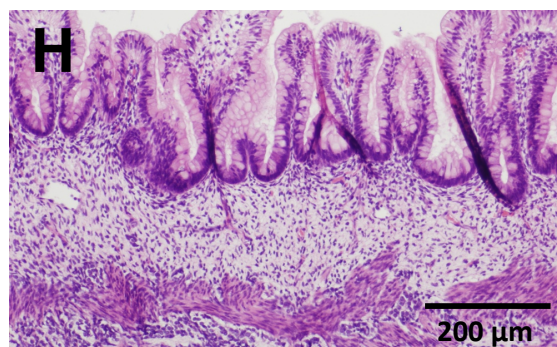

**HiPS-  
RIKEN-  
1A**

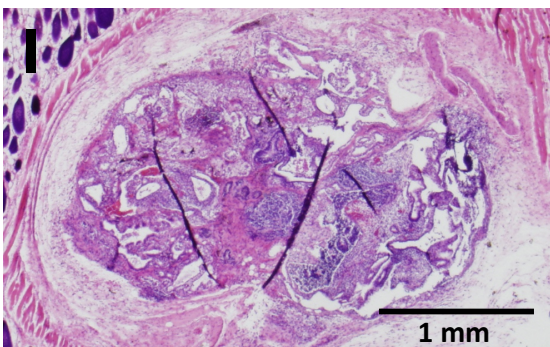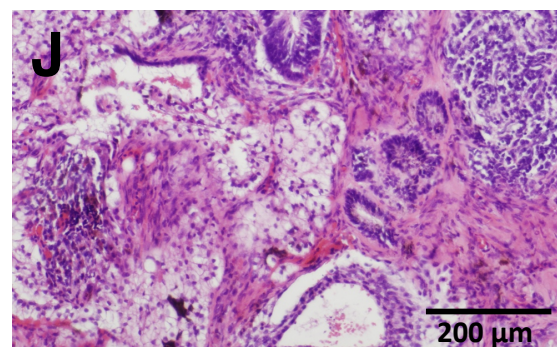

**HiPS-  
RIKEN-  
2A**

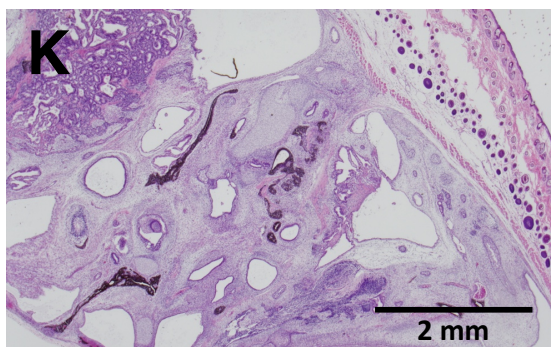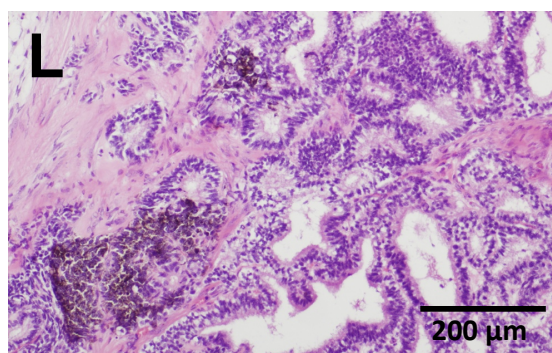

**HiPS-  
RIKEN-  
12A**

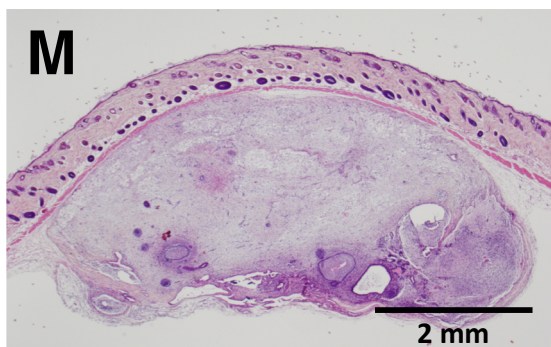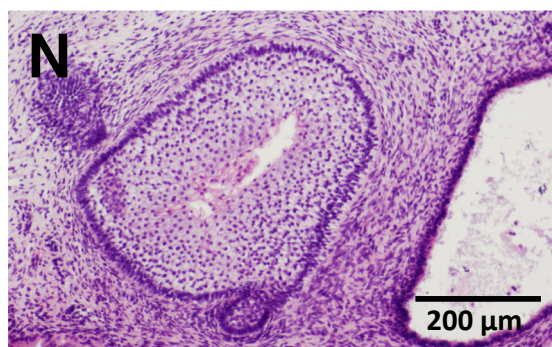

**ATCC-  
DYR  
0100**

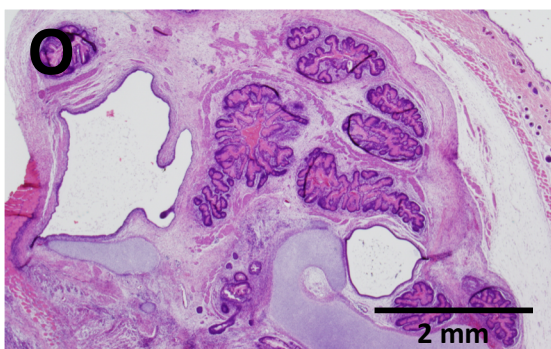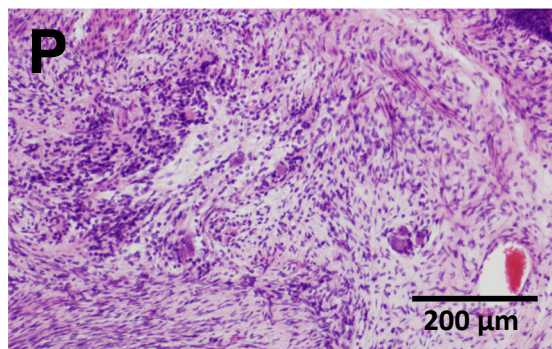

**ATCC-  
HYR  
0103**

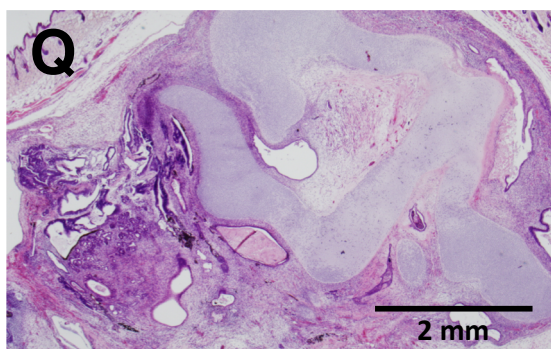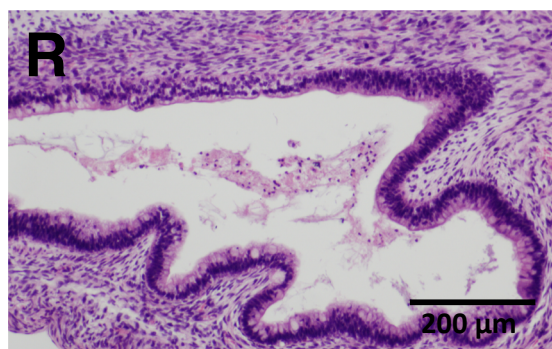

**mc-iPS**

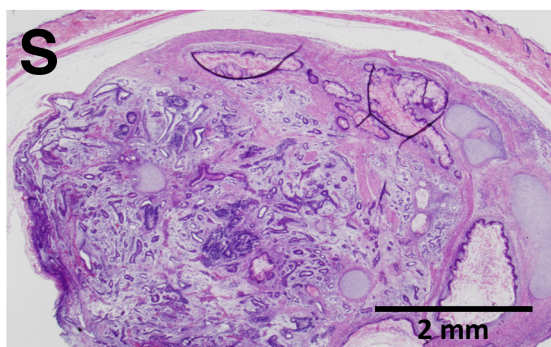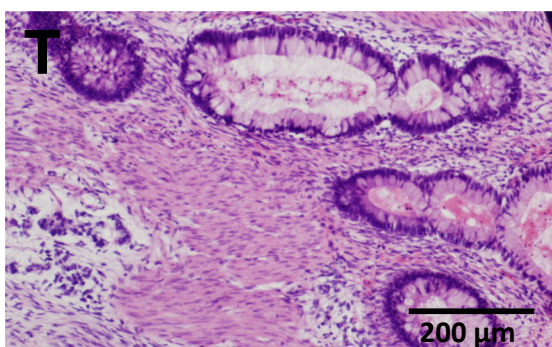

Supplement: S1 Fig — NOG mice were subcutaneously injected with 3 × 10^4^ hiPSCs in a mixture of Matrigel, 1 × 10^6^ mitomycin C-treated NHDF, and 10 μM Y-27632. Typical images of teratomas derived from 10 hiPSC lines are shown stained with hematoxylin and eosin [201B7 #3 (A, B), 253G1 #6 (C, D), 409B2 #3 (E, F), 454E2 #6 (G, H), HiPS-RIKEN-1A #6 (I, J), HiPS-RIKEN-2A #6 (K, L), HiPS-RIKEN-12A #5 (M, N), ATCC-DYR0100 #6 (O, P), ATCC-HYR0103 #4 (Q, R), and mc-iPS #5 (S, T)]. Low power view (× 1.25) of teratoma represents two or three germ layer components (A, C, E, G, I, K, M O, Q and S). Higher power view (× 10) shows mesodermal cartilage and endodermal intestinal tract-like duct (B), ectodermal glial tissues, melanocytes and choroid-like tissues (D), ectodermal glial tissues (F), mesodermal smooth muscle and endodermal intestinal tract-like duct (H), ectodermal choroid-like tissues and immature neuroepithelia (J), ectodermal choroid-like tissues and melanocytes (L), ectodermal stratified squamous epithelia and endodermal duct structures (N), ectodermal glial and neural cells and mesodermal blood vessels (P), endodermal duct structures accompanied with intestinal and respiratory epithelium-like cells (R), and mesodermal smooth muscle and endodermal intestinal tract structures (T). (PDF) [file pone.0205022.s008.pdf]

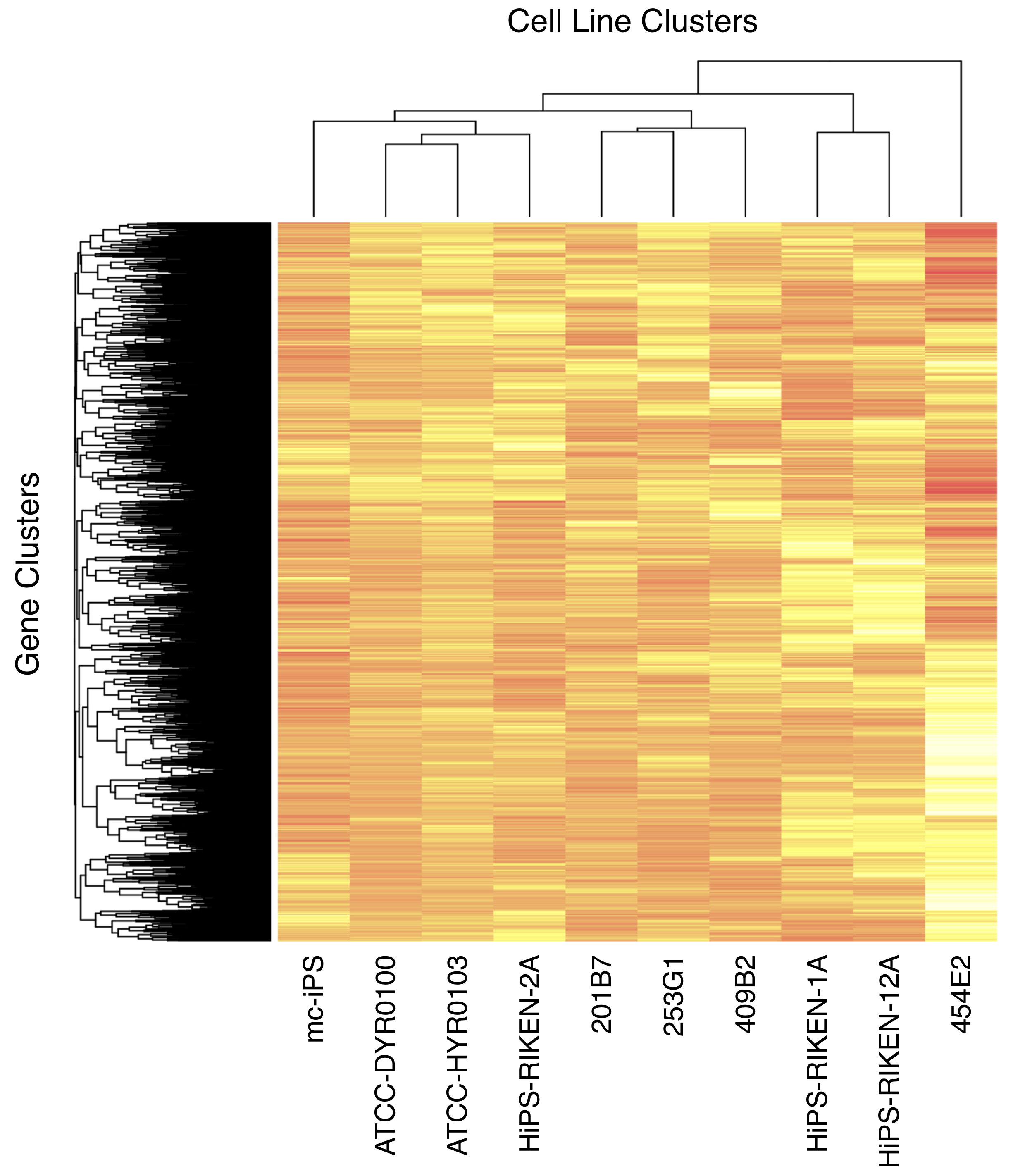

Supplement: S2 Fig — A set of 16,454 probes on GeneChip Human Genome U133 Plus 2.0 Array was statistically identified with significantly different expression levels among 10 hiPSC lines (one-way ANOVA, p < 0.05). Hierarchical clustering analysis was performed using R version 3.5.1 software. (TIFF) [file pone.0205022.s009.tiff]

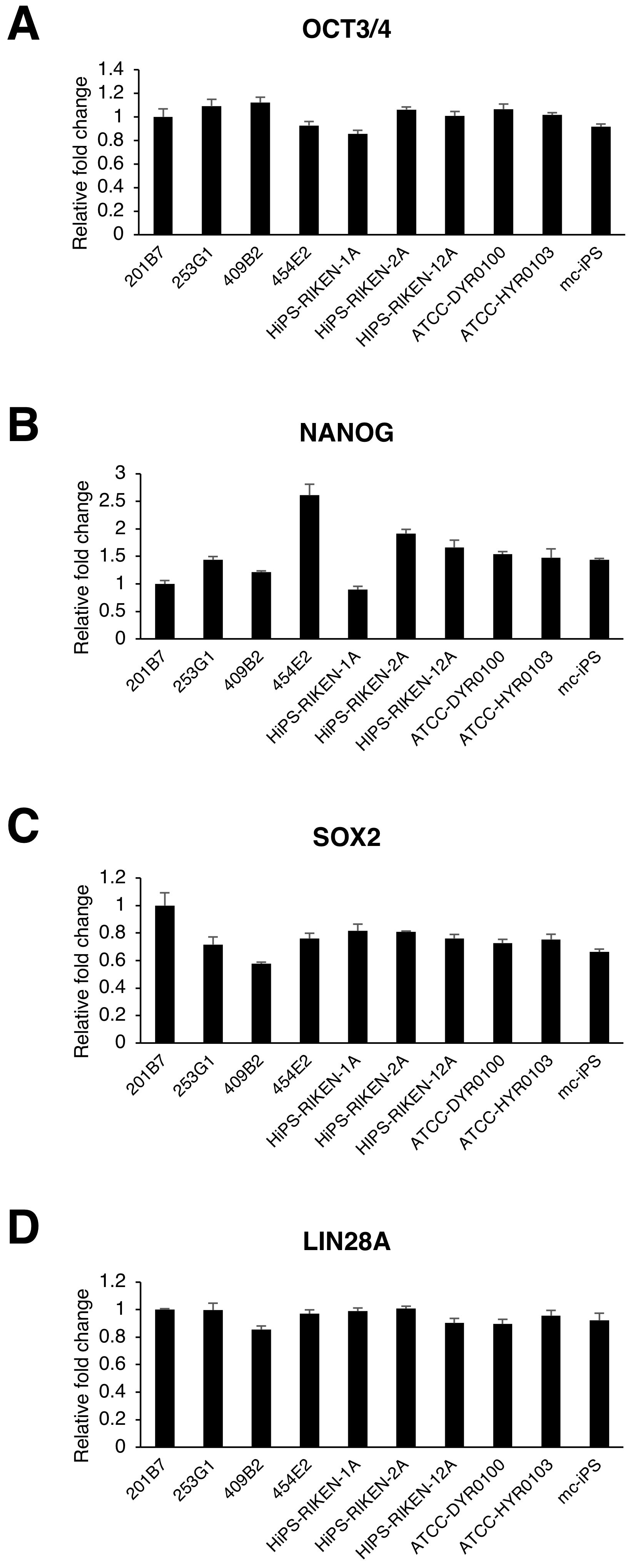

Supplement: S3 Fig — Transcript expression of OCT3/4 (A), NANOG (B), SOX2 (C), and LIN28A(D) in 10 hiPSC lines is shown with microarray data. Data are represented as mean ± SD (n = 3). (TIFF) [file pone.0205022.s010.tiff]
